# Mutation of putative glycosyl transferases PslC and PslI confers susceptibility to antibiotics and leads to drastic reduction in biofilm formation in *Pseudomonas aeruginosa*

**DOI:** 10.1099/mic.0.001392

**Published:** 2023-09-13

**Authors:** Rohit Ruhal, Moumita Ghosh, Vineet Kumar, Deepti Jain

**Affiliations:** ^1^​ Transcription Regulation Lab, Regional Centre for Biotechnology, NCR Biotech Science Cluster, 3rd Milestone, Faridabad-Gurgaon Expressway, Faridabad, 121001, India

**Keywords:** glycosyl transferase, PslC, PslI, *Pseudomonas aeruginosa*, biofilm

## Abstract

*
Pseudomonas aeruginosa* is an opportunistic, multidrug-resistant pathogen capable of adapting to numerous environmental conditions and causing fatal infections in immunocompromised patients. The predominant lifestyle of *P. aeruginosa* is in the form of biofilms, which are structured communities of bacteria encapsulated in a matrix containing exopolysaccharides, extracellular DNA (eDNA) and proteins. The matrix is impervious to antibiotics, rendering the bacteria tolerant to antimicrobials. *P. aeruginosa* also produces a plethora of virulence factors such as pyocyanin, rhamnolipids and lipopolysaccharides among others. In this study we present the molecular characterization of *pslC* and *pslI* genes, of the exopolysaccharide operon, that code for putative glycosyltransferases. PslC is a 303 amino acid containing putative GT2 glycosyltrasferase, whereas PslI is a 367 aa long protein, possibly functioning as a GT4 glycosyltransferase. Mutation in either of these two genes results in a significant reduction in biofilm biomass with concomitant decline in c-di-GMP levels in the bacterial cells. Moreover, mutation in *pslC* and *pslI* dramatically increased susceptibility of *P. aeruginosa* to tobramycin, colistin and ciprofloxacin. Additionally, these mutations also resulted in an increase in rhamnolipids and pyocyanin formation. We demonstrate that elevated rhamnolipids promote a swarming phenotype in the mutant strains. Together these results highlight the importance of PslC and PslI in the biogenesis of biofilms and their potential as targets for increased antibiotic susceptibility and biofilm inhibition.

## Introduction

*Pseudomonas aeruginosa* is a Gram-negative pathogen capable of thriving and adapting to myriad environmental conditions. It is responsible for acute and chronic infections in humans and has become a serious public health threat. It infects patients with burn wounds, cystic fibrosis, ventilator-associated pneumonia, etc. It also results in device-related infections particularly in immunocompromised patients [1]. *P. aeruginosa* has a remarkable ability to switch from a motile to sessile phenotype where it adheres to biotic or abiotic surfaces. This transition triggers the production of exopolysaccharides leading to the formation of biofilms, which are structured communities of bacteria encapsulated in self-produced matrix, primarily composed of extracellular polymeric substances (EPS) [2]. Biofilm-related infections pose a significant challenge for eradication as they provide protection against antimicrobials and the host defence system [3]. The exopolysaccharides within the biofilm matrix play a vital role in adhesion, stability and structural integrity [4]. Eradication of *P. aeruginosa* is difficult due to tolerance towards a wide variety of antimicrobial agents due to its biofilm-forming capacity [5]. Consequently, there is an urgent need for characterization of biofilm-related novel targets.

The composition of the biofilm matrix differs significantly between different microbes. In case of *P. aeruginosa* it is largely composed of three different types of exopolysaccharides, Psl, Pel and alginate [6]. Alginate is a negatively charged polymer of guluronic and mannuronic acid and is responsible for the mucoid phenotype of *P. aeruginosa* while non-mucoid strains produce Psl and Pel [7]. Pel is a positively charged polymer composed of amino sugars α-1,4 linked *N*-acetylgalactosamine and galactosamine repeat crosslinked to eDNA. It contributes towards cell–cell interactions and the biomechanical properties of biofilms [8–10]. The Psl polysaccharide, on the other hand, is a neutral polysaccharide consisting of repeating pentamers of d-glucose, d-mannose and l-rhamnose [11]. It is critical for surface attachment during the initial stages of biofilm formation and is essential for maintaining the structure of mature biofilms [12]. Additionally, Psl acts as a signalling molecule that regulates the production of exopolysaccharide by stimulating the production of c-di-GMP [13].

The gene cluster designated as *psl* in the genome of *P. aeruginosa* PAO1 comprises 15 co-transcribed genes (*pslA* to *pslO*) [14]. In-frame deletion of individual genes revealed that 11 of these cause drastic reduction in biofilm formation [15]. Four of these genes, *pslC*, *pslF*, *pslH* and *pslI*, code for putative uncharacterized glycosyl transferases [16]. These enzymes aid in the transfer of the sugar moiety from the activated nucleotide-sugar to an acceptor. Transcriptional control of Psl synthesis is exerted by FleQ. During the planktonic stage of the bacteria, FleQ represses *psl* transcription but this repression is relieved upon binding of c-di-GMP to FleQ, and the associated conformational changes permit FleQ to activate the expression of *psl* [17, 18]. Diguanylate cyclases (DGCs) and phosphodiesterases (PDEs) regulate the levels of c-di-GMP in the cells [19]. An increase in the intracellular concentration of c-di-GMP triggers Psl biosynthesis and leads to biofilm formation whereas lower concentrations promote biofilm dispersal and motility [20].

Biofilm formation in *P. aeruginosa* is also regulated through quorum sensing (QS). QS is also referred to as the bacterial cell to cell communication signalling pathway [21, 22]. The QS system is activated when autoinducers bind to their cognate receptors which results in alterations in gene expression. Four QS cascades have been characterized in *P. aeruginosa *: LasI/LasR, RhlI/RhlR, PqsR and QscR [23–25]. In addition to biofilm formation, these pathways regulate the generation of various virulence factors such as pyocyanin, elastase, exotoxin A, rhamnolipids, lectins, etc. [26, 27]. Rhamnolipids are vital for maintaining channels for nutrient distribution within biofilms [28, 29]. The QS pathway also positively regulates the production of pyocyanin, a phenazine molecule, at the transcriptional level. LasR, when bound to its inducer, *N*-3-oxo-dodecanoyl-homoserine lactone, activates the expression of RhlR. RhlR in complex with its autoinducer *N*-butyryl-homoserine lactone activates the gene responsible for pyocyanin production [30]. The synthesis of this pigment is also regulated at the post-transcriptional level by the Rsm pathway [31].

Few classes of antibiotics are effective against the planktonic form of *P. aeruginosa *. However, most are ineffective against the biofilms because of the impervious nature of the exopolysaccharide matrix making them 1000-fold more resistant [32]. It has been demonstrated that Psl confers biofilm-associated tolerance towards antibiotics such as tobramycin, colistin and ciprofloxacin [33, 34]. A detailed understanding of the molecular mechanism of biofilm formation and associated antibiotic resistance will provide crucial intervention points against persistent infections. In the current study we focused on the role of PslC and PslI, two known glycosyltransferases [16]. Our results indicate that mutation in these genes not only affects Psl biosynthesis but also impacts different phenotypes including antibiotic susceptibility, motility, biofilm formation, intracellular c-di-GMP concentration and rhamnolipid production. We show that loss of *pslC* and *pslI* genes results in a drastic reduction in biofilm biomass with simultaneous reduction in c-di-GMP levels within the bacterial cells. Thus, these proteins are required for the synthesis of the biofilm matrix. Further, our data show that the mutants are susceptible to antibiotics and show increased swarming motility, pyocyanin and rhamnolipid production.

## Methods

### Bacterial strains and growth conditions

*P. aeruginosa* PAO1 (wild-type) and mutants of *pslC* (PW4800), *pslI* (PW4809) and *rhlI* (PW6880) were procured from the mutant library maintained in Manoil lab. The mutants were prepared by insertion of the ISphoA/hah transposon at positions 380, 1098 and 125 in the respective ORFs [35]. All the mutants were confirmed by PCR. The *pslC* and *pslI* mutants are denoted as *Tn-pslC* and *Tn-pslI*. For complementation, the *pslC* and *pslI* genes were subcloned in pHERD20T plasmid. For *in vivo* experiments, pHERD20T*-pslC*, pHERD20T*-pslI* and empty plasmid were transformed in the respective mutant strains by electroporation and selected against 300 µg ml^−1^ carbenicillin. The strains were cultured at 37 °C in lysogeny broth (LB) and cell density was determined by measuring the optical density at a wavelength of 600 nm.

### Biofilm assay

Biofilm formation was performed in glass test tubes [36]. The overnight culture was diluted to an OD_600_ of 0.05 containing 1×10^8^ cells ml^−1^ in LB. Then, 1000 µl of the diluted culture was transferred to glass tubes in triplicate supplemented with 0.3 % (w/v) arabinose. The tubes were then incubated for 24 h at 37 °C in static condition. After 24 h of incubation the free-floating bacterial cells were removed and tubes were washed twice with sterile water and allowed to dry for 30 min. The attached biomass was stained with 0.1 % crystal violet for 15–20 min at room temperature and extra stain was discarded. The biomass was washed three times with sterile water. For quantification, the bound crystal violet was dissolved in 30 % glacial acetic acid and absorbance was measured at 595 nm in a platereader (Spectramax M5). The experiment was repeated three times, each time in triplicate.

### Growth curve assay

To examine the growth of planktonic cells of wild-type, mutant and complemented *P. aeruginosa *, growth curve analysis was performed. Cultures were grown overnight in LB at 37 °C at 180 r.p.m. and diluted to an OD_600_ of 0.05 such that the number of cells was 1×10^8^ cells ml^−1^. Then 200 µl of diluted culture was transferred to a 96-well microtitre plate in triplicate and incubated for 16 h at 37 °C. The OD at 600 nm was measured every hour for 16 h and normalized by LB readings.

### Confocal microscopy

To observe mature biofilm structures of the strains, a live/dead bacterial viability kit was used. Mid-log phase cells with colony forming units (c.f.u.) of 1×10^8^ cells ml^−1^ were allowed to grow on a glass coverslip in a six-well plate for 24 h. After 24 h, free floating planktonic cells were removed carefully and 2 ml fresh LB medium was added and incubated again for 24 h at 37 °C. LB was supplemented with 300 µg ml^−1^ carbenicillin and 0.3 % (w/v) arabinose for complemented and empty vector control (VC) strains. After 48 h of incubation, the planktonic cells were removed gently and washed with sterile water twice. For staining, Syto9 (3.4 mM) and propidium iodide (PI) (20 mM) mixed in sterile mQ water were added at a ratio of 7 : 3 ratio in the wells and incubated at 37 °C for 20 min in the dark. The stained coverslips were then inverted slowly on oil immersion onto a glass slide and fixed. The slides were then visualized in a confocal microscope (Laser Scanning Confocal Microscope: LSM 880; Carl Zeiss) under a 63× oil objective lens at a wavelength 488 nm for SYTO9 and 543 nm for PI. The percentage of viable cells was calculated using Imaris 8.1 software.

### Antibacterial susceptibility tests

To examine sensitivity of the mutants towards antibiotics, disc diffusion assay was carried out. Fresh overnight bacterial cultures diluted to approximately 10^8^ cells ml^−1^ were swabbed evenly on an MHA (Mueller Hinton agar) plate and left to dry for 10 min. Discs containing antibiotics, tobramycin (10 µg), ciprofloxacin (5 µg) and colistin (10 µg), were placed carefully at the centre of the plate using a sterile forceps and then plates were incubated overnight at 37 °C. Next day, the inhibition zone was measured. Culture was also spread-plated with no antibiotic as a control. Three independent experiments were performed.

### Determination of minimum inhibitory concentrations

To calculate MICs, cultures of wild-type PAO1 and mutants were grown from freshly streaked MHA plates. A gradient of two-fold dilutions of antibiotics ranging from 512 to 1 µg ml^–1^ were prepared in MHB. One hundred microlitres of antibiotics was mixed with 100 µl of bacterial culture such that the final concentration of cells was 5×10^5^ cells ml^−1^. After mixing, the plate was incubated overnight at 37 °C and absorbance at 600 nm was measured to calculate the MIC of antibiotics. The OD_600_ values were normalized with the values of only media control wells.

### Determination of minimum biofilm eradication concentration (MBEC)

To calculate MBEC, a 24 h biofilm was allowed to form in sterile 96-well round-bottom plates at 37 °C. The next day the plate was inverted to remove free floating cells and washed twice with sterile water. Two-fold dilutions of antibiotics ranging from 512 to 1 µg ml^−1^ were prepared in LB. Then, 200 µl of each concentration was added in the corresponding well in duplicate and incubated at 37 °C overnight. After incubation, the method of O’Toole was used to quantify biofilm biomass as described above [37].

### Determination of swimming motility

This assay was performed as described earlier [17]. Briefly, single colonies were poked mid-way into a 0.3 % LB agar plate using a sterile toothpick and plates were incubated at 37 °C for 14 h. The diameter of the motility zone was measured next day. Three independent experiments were performed to determine the statistical significance.

### Determination of swarming motility

Swarming motility was determined in 0.5 % nutrient agar plates containing 5 g l^−1^ glucose. Plates with equal depth were prepared and dried under laminar flow for 30 min. Three microlitres of fresh mid-exponential phase culture was placed in the centre of each plate, dried and incubated at 37 °C for 14 h. The diameter was measured the next day and plotted.

### Determination of twitching motility

A subsurface stab assay was performed to determine the twitching motility of the samples in which mid-exponential phase cells were stabbed mid-way into a 1.5 % LB agar plate. The size of the twitching zone was measured after incubation at 37 °C overnight.

### Quantification of rhamnolipid

For rhamnolipid quantification, 2 ml of a 48 h grown culture, each of wild-type PAO1, *pslC* and *pslI* mutants was centrifuged for 10 min at 6000 *g* and supernatant was concentrated to half the volume. 100 µl of concentrated HCl was added until the pH reached 2 followed by addition of an equal volume of chloroform/methanol mixture and vortexed for 1 min. The lower organic phase was collected and used for rhamnolipid quantification. An anthrone calorimetric assay was used to measure rhamnolipid in culture and a rhamnose standard curve was used to obtain the concentration.

### Drop collapse assay

A 2 ml culture of wild-type and mutants after 2 days of incubation at 37 °C at 180 r.p.m. in LB was centrifuged at 6000 ***g*** for 10 min to remove bacterial culture. The supernatant was filtered and collected in a fresh tube concentrated to half the volume. 20 µl of each culture were spotted in triplicate onto the lid of a 96-well plate to observe bead formation. Samples that did not form a bead were characterized as collapsed drops.

### Quantification of pyocyanin

Cultures of wild-type *P. aeruginosa* PAO1 and transposon mutants of *Tn-pslC* and *Tn-pslI* grown for 48 h were centrifuged to obtain cell-free supernatant. 2 ml of supernatant was concentrated to 500 µl and an equal volume of chloroform was added, and the mixture was vigorously vortexed for 5 min until the it turned blue-green colour. For phase separation, the mixture was centrifuged at 3000 ***g*** for 10 min at 4 °C. The lower blue coloured pyocyanin was extracted and mixed with 200 µl of 0.1 M concentrated HCl and vortexed for 5 min. The blue layer turns pink after acidification followed by phase separation. The upper pink phase containing complete extracted pyocyanin was collected and absorbance was measured at 500 nm. Measurement was normalized to the media control. The experiment was repeated independently three times each in triplicate.

### c-di-GMP measurement

To measure c-di-GMP levels inside the free-floating bacterial cells, a plasmid-based reporter assay was performed [38]. Briefly, reporter plasmid pCdrA::gfp^C^ was introduced into *P. aeruginosa* wild-type PAO1, *Tn-pslC*, *Tn-pslI* and complemented strains by electroporation. These transformed cells, designated as PAO1/pCdrA::gfp^C^, *Tn-pslC/pCdrA::gfp^C^*, *Tn-pslI/pCdrA::gfp^C^*, *Tn-pslC::pslC/pCdrA::gfp^C^* and *Tn-pslI::pslI/pCdrA::gfp^C^*, were used to measure c-di-GMP *in vivo*. The strains were inoculated in LB from fresh streaks on LB agar supplemented with 60 µg ml^−1^ gentamicin and 300 µg ml^−1^ carbenicillin overnight. Overnight grown cells were diluted to an OD of 0.05 in 5 ml LB supplemented with 60 µg ml^−1^ gentamicin, 300 µg ml^−1^ carbenicillin and 0.3 % (w/v) arabinose and grown for 24 h at 37 °C. Fluorescence was measured as relative fluorescence intensity units (RFUs) using a spectrophotometer with 490 nm excitation filter and 515 nm emission filter. Measurement was normalized to the LB control.

### Modelling

The coordinates of PslC and PslI models were obtained from the AlphaFold2 database for *P. aeruginosa* (https://alphafold.ebi/ac.uk/). The structural homologues of PslC and PslI were searched in DALI (http://ekhidna2.biocenter.helsinki.fi/dali/). Superimposition, docking of ligands and structural analysis was carried out in PyMol (http://www.pymol.org/pymol).

## Results

### PslC and PslI are putative glycosyltransferases

**Fig. 1.**
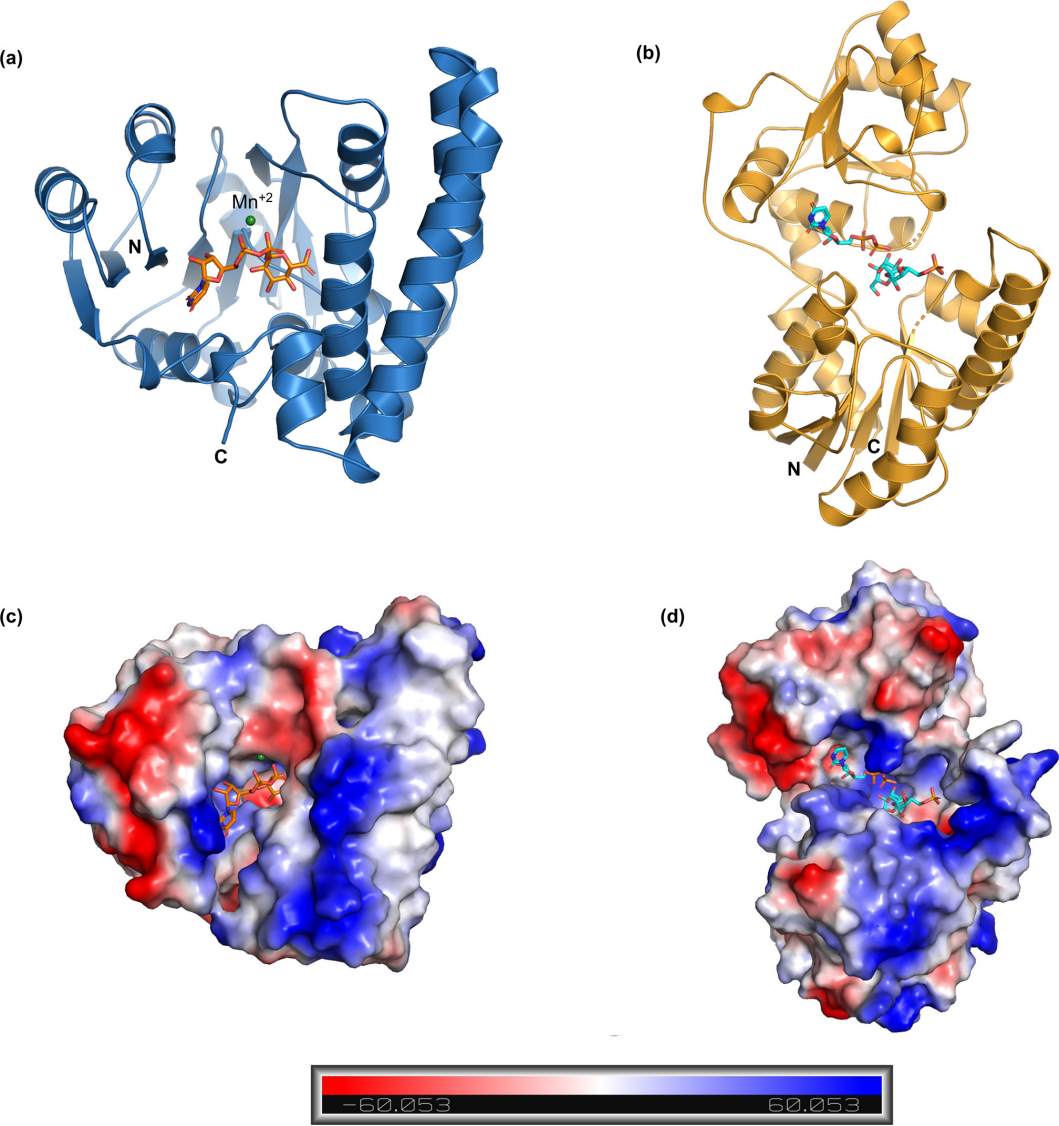
Model of PslC and PslI. (**a**) AlphaFold2 model of PslC (blue ribbon). The substrate UDP-glucuronic acid and Mn^2+^ ion is docked in the active site from the PDB: 2Z86. (**b**) AlphaFold2 model of PslI (gold ribbon) . The ligands, UDP and sucrose-6-phosphate are docked from the PDB: 6KIH. (c, d) Electrostatic surface potential of PslC and PslI coloured according to the bar beneath, highlighting the ligands bound in the potential catalytic site.

A blast search revealed that PslC (303 aa) and PslI (367 aa) do not show any significant sequence homology to proteins whose structures have been determined and deposited in the protein data bank (PDB) but placed PslC in the glycosyltransferase GT2 family and PslI in the GT4 family of proteins. Structure prediction using PSIPRED revealed well-ordered regions for both proteins (Fig. 1a, b). We then modelled PslC and PslI using AlphaFold2.

The AlphaFold2 model of PslC shows that the enzyme has a glycosyltransferase-A fold. Comparison of the PslC structure with other structures in the PDB using a DALI search revealed that the model exhibited highest structural similarity with mannosyl transferase from *Pyrobaculum calidifontis* (PcManGT) (PDB:6YV7) (Table S1, available in the online version of this article) [39]. While PcManGT has a transmembrane region composed of three antiparallel helices, this region is absent in PslC indicating that PslC is possibly a soluble enzyme. The structure is composed of a core of a β-sheet of eight strands surrounded by nine α-helices. The docking of substrate UDP-GlcUA (UDP-glucuronic acid) on the PslC model from the structure of chondroitin polymerase from *Eschericha coli* (PDB: 2Z86) shows that there is a tight fit of the substrate in the active site (Fig. 1a, c) [40].

Structural analysis of PslI shows that it is a two-domain protein with both the N- and C-terminal domain with the Rossmann-type fold. The two domains have high structural homology despite very weak sequence similarity. A deep cleft that separates the two domains constitutes the catalytic centre. Two chain segments connect the two domains allowing a high degree of flexibility. The last α-helix harbours a kink, a conserved feature in this family of enzymes. A DALI search using the AlphaFold2 model against the PDB revealed that PslI displays the highest structural similarity with sucrose phosphate synthase bound to UDP and sucrose 6-phosphate (S6P) from *Thermosynechococcus elongates* (PDB: 6KIH) (Table S2) [41]. The members of this family of proteins are involved in the transfer of sugar moieties from activated donor molecules to specific acceptor molecules resulting in glycosidic bond formation. Superimposition of the ligands, UDP and S6P from PDB: 6KIH onto the PslI model shows that while the S6P is bound towards the N-terminal domain, the donor sugar binds towards the C-terminal domain. The electrostatic potential surface of the PslI model is depicted in Fig. 1(d).

### Mutation of *pslC* and *pslI* leads to a reduction in biofilm formation

**Fig. 2.**
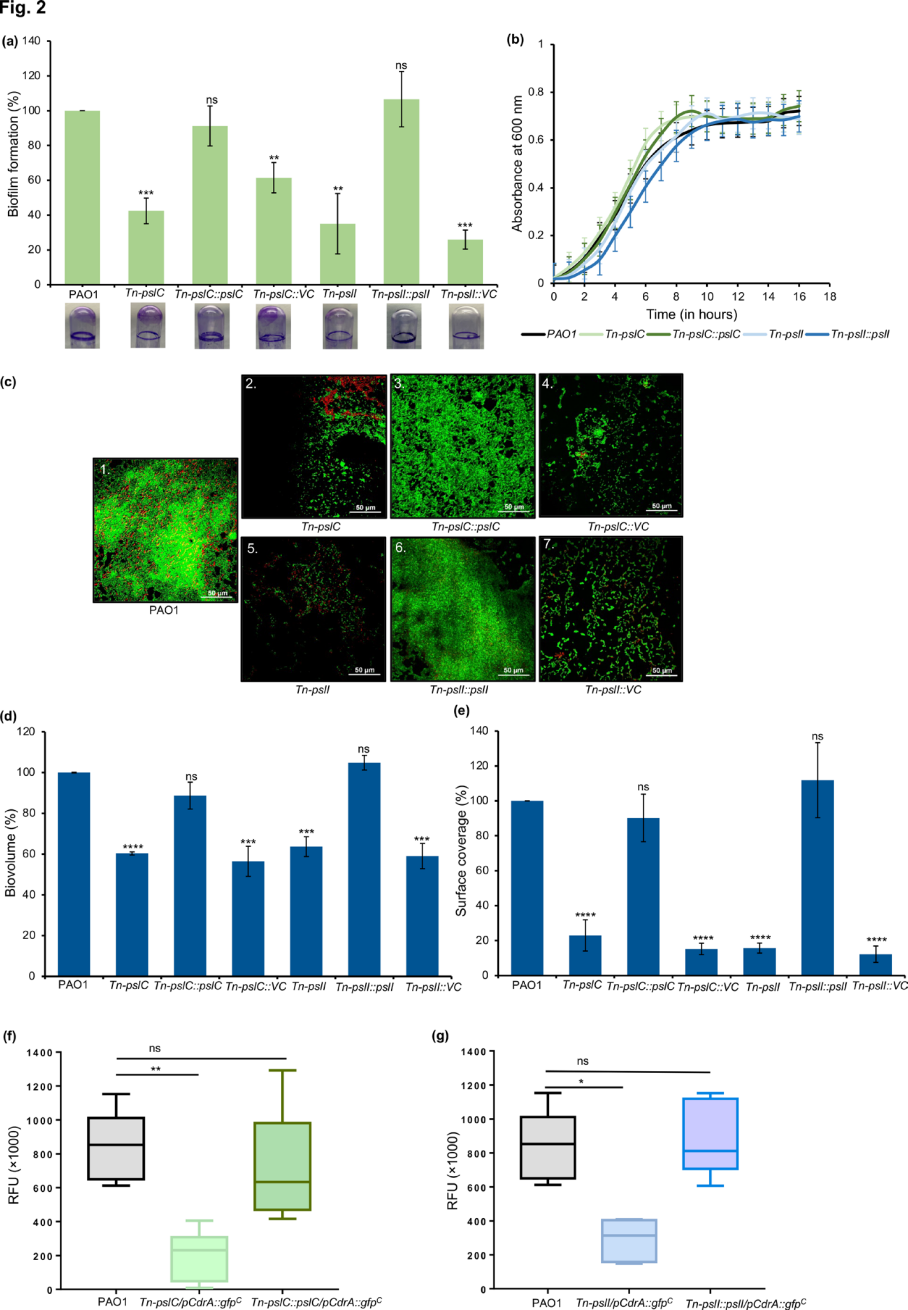
Effect of transposon mutant *pslC* (*Tn-pslC*) and *pslI* (*Tn-pslI*) on biofilm biomass. (**a**) Percentage of biofilm biomass formed by wild-type PAO1, mutants and mutants complemented with *pslC* (*Tn-pslC::pslC*), *pslI* (*Tn-pslI::pslI*) and vector control (*Tn-pslC::VC* and *Tn-pslI::VC*) plotted as a bar diagram. A representative image of glass tubes with biofilm formed is shown under each corresponding column. (**b**) Growth curve of *P. aeruginosa* wild-type PAO1, *Tn-pslC* and *Tn-pslI* mutants as well as respective complemented strains. (**c**) Confocal laser scanning microscopy images of biofilm biomass of (1) wild-type PAO1, (2) *Tn-pslC,* (3) *Tn-pslC::pslC,* (4) *Tn-pslC::VC,* (5) *Tn-pslI,* (6) *Tn-pslI::pslI* and (7) *Tn-pslI::VC* stained using BacLight Live/Dead stain. Live cells are stained with Syto9 (green) and dead cells with propidium iodide (red). (**d**) Percentage of total biovolume with respect to wild-type PAO1, determined after 48 h of biofilm formation on glass coverslips. (**e**) Percentage of biofilm surface coverage of mutants and complemented strains with respect to wild-type PAO1. Quantification of c-di-GMP in wild-type PAO1/pCdrA::gfp^C^ (**f**) *Tn-pslC/pCdrA::gfp^C^* and *Tn-pslC::pslC/pCdrA::gfp^C^* and with (**g**) *Tn-pslI/pCdrA::gfp^C^* and *Tn-pslI::pslI/pCdrA::gfp^C^* strains of *P. aeruginosa* under planktonic conditions by measuring relative fluorescence intensity units (RFU) at 24 h. All experimental data are the mean of three independent experiments. Statistical significance (*P≤0.05; **P≤0.01; ***P≤0.001; ****P≤0.0001) was determined using an unpaired Student’s *t*-test.

The *psl* operon has been shown to be essential for biofilm formation and PslC and PslI are putative glycosyl transferases and play an important function in Psl production. A decrease in Psl production would lead to reduced biofilm formation. We first sought to examine the biofilm forming ability of transposon mutants of *pslC* (*Tn-pslC*) and *pslI* (*Tn-pslI*) using the crystal violet assay. A significant reduction (60–65 %) in biofilm formation was observed based on total biomass calculated as compared to the wild-type PAO1 (Fig. 2a). This is consistent with an earlier study [15]. Further, complementation of *pslC* (*Tn-pslC::pslC*) and *pslI* (*Tn-pslI::pslI*) integrated on the plasmid pHERD20T restores the biofilm-forming capacity to wild-type levels (Fig. 2a). On the other hand, complementation with empty vector (*Tn-pslC::VC* and *Tn-pslI::VC*) showed no difference (Fig. 2a). These results show that *pslC* and *pslI* gene products are essential for biofilm formation.

Further, a growth assay was carried out to determine if the reduction in biomass is due to changes in growth of the mutant strains (Fig. 2b). Growth of the wild-type PAO1, two mutants and the complemented strains was monitored in LB liquid medium at 37 °C for 16 h. No significant difference in growth of the *Tn-pslC* and *Tn-pslI* mutants or the complemented strains was observed compared to wild-type PAO1 confirming that the difference in biofilm formation was not due to a difference in growth of the wild-type and mutants.

To determine if the reduction in biofilm biomass was due to reduced exopolysaccharide matrix in the mutants, biofilm was also evaluated using confocal laser scanning microscopy (CLSM). While the parent strain PAO1 formed dense biofilms, dramatically reduced biofilms were formed in *Tn-pslC* and *Tn-pslI* (Fig. 2c). Mutant biofilms had greatly reduced matrix and showed less defined microcolonies. Further, the percentage of biovolume and surface coverage was determined using IMARIS software [42]. The *Tn-pslC* and *Tn-pslI* mutants showed about 40 and 30% reduction in biovolume compared to the parent strain respectively (Fig. 2d). The surface coverage of both the mutants was reduced significantly as compared to wild-type PAO1 (Fig. 2e). Complementation of the mutant strains with their respective genes restored the wild-type phenotype in terms of quality of biofilm formed, biovolume and surface coverage of the biofilms (Fig. 2c, d, e). Complementation with the empty vector (VC) had no effect. This implies that PslC and PslI are key enzymes for biosynthesis of exopolysaccharide, a component of the matrix of the *P. aeruginosa* biofilms.

### The *pslC* and *pslI* mutation led to lowering of c-di-GMP concentration

The level of secondary messenger c-di-GMP in bacterial cells is a major determinant for biofilm formation. Since Psl acts as a signalling molecule that regulates the production of c-di-GMP, we next measured the intracellular levels of c-di-GMP in the wild-type PAO1, *Tn-pslC* and *Tn-pslI* using a fluorescence-based reporter assay [38]. PAO1 showed a stronger GFP fluorescence signal, but levels of the secondary messenger molecule reduced by 4-fold in *Tn-pslC* (Fig. 2f) and 2.9-fold in case of *Tn-pslI* (Fig. 2g) compared to wild-type PAO1. Complementation of the respective glycosyltransferases revealed similar fluorescence intensity as the wild-type, indicating that the levels of secondary messenger in the bacterial cells was restored.

Taken together, our results demonstrate that mutation of *pslC* or *pslI* causes a defect in biofilm formation and reduction in the intracellular levels of c-di-GMP.

### Mutation in *pslC* or *pslI* leads to increased susceptibility to antibiotics in planktonic form

We next tested susceptibility of the mutants to three different antibiotics, tobramycin, colistin and ciprofloxacin belonging to three different classes, in the planktonic form of *P. aeruginosa *. While tobramycin is an aminoglycoside, colistin is a polymyxin cationic antimicrobial peptide and ciprofloxacin is a fluoroquinolone [33].

**Fig. 3.**
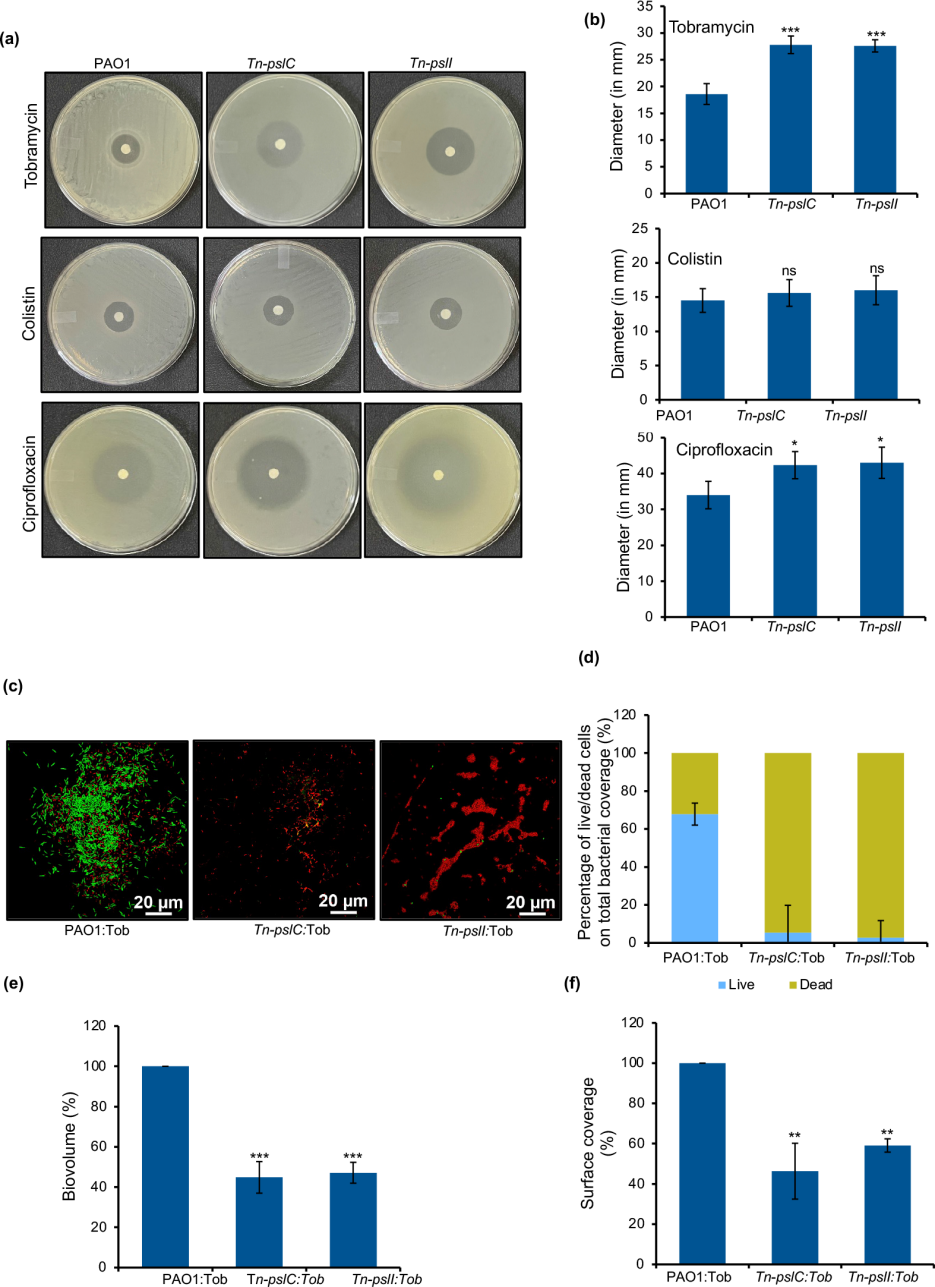
Antibiotic susceptibility. (**a**) Images of wild-type PAO1 and mutants of *pslC* (*Tn-pslC*) and *pslI* (*Tn-pslI*) on MHA plates. (**b**) Plot showing the diameter of zone of inhibition against antibiotics for the wild-type and mutants. (**c**) Representative CLSM images of PAO1 and mutants after treatment with 4 µg ml^−1^ tobramycin. (**d**) Percentage of live/dead cells in biofilm. (**e**) Percentage of total biovolume and (**f**) surface coverage for PAO1 and mutants after treatment with tobramycin determined and quantified using BacLight Live Dead stain and calculated using IMARIS 8.1 software. Error bar represents se (*n*≥3). **P ≤ 0.01; ***P ≤ 0.001; statistical significance determined by an unpaired Student’s *t*-test.

We found that *Tn-pslC* and *Tn-pslI* exhibited higher susceptibility to tobramycin and ciprofloxacin in disc diffusion assays (Fig. 3a, b). Colistin did not show any significant difference possibly due to less diffusion of the antibiotic (Fig. 3a, b) [43]. We next quantified the MIC of all three antibiotics in the planktonic form of PAO1 and the two mutants. The MIC of the parental PAO1 strain was two-fold higher compared to *Tn-pslC* and *Tn-pslI* for tobramycin and ciprofloxacin and was four-fold higher for colistin (Table 1). Thus, mutation in any of the two glycosyl transferases leads to susceptibility to antibiotics in the planktonic form of bacterial growth.

**Table 1.** The MIC (minimum inhibitory concentration) and MBEC (minimum biofilm eradication concentration) of tobramycin, colistin and ciprofloxacin for wild-type and *Tn-pslC* and *Tn-pslI* mutants

	MIC (µg ml^–1^)			MBEC (µg ml^–1^)		
	Tobramycin	Colistin	Ciprofloxacin	Tobramycin	Colistin	Ciprofloxacin
PAO1	1	2	0.25	16	64	4
*Tn-pslC*	0.5	0.5	0.12	4	8	1
*Tn-pslI*	0.5	0.5	0.12	4	8	2

### The *pslC* or *pslI* mutation leads to increased susceptibility of biofilm to antibiotics

We also tested the susceptibility of biofilms formed by the wild-type PAO1 and the mutants to all three antibiotics. We determined changes in MBEC upon mutation in *pslC* and *pslI*. The susceptibility of *pslC* and *pslI* mutants increased as the minimum concentration required for eradication of biofilms (MBEC) came down to 4 and 8 µg ml^−1^ compared to 16 and 64 µg ml^−1^ in the parent strain for tobramycin and colistin respectively (Table 1). The MBEC for ciprofloxacin for *Tn-pslC* and *Tn-pslI* fell to 1 and 2 µg ml^−1^ respectively from 4 µg ml^−1^. Thus, mutation of glycosyl transferases makes the *P. aeruginosa* biofilms susceptible to a range of antibiotics.

Biofilm formation was also evaluated using confocal microscopy in the presence of tobramycin by live/dead staining. Live bacteria with an intact membrane were stained with SYTO9 and are depicted in green whereas non-viable bacteria with a damaged membrane incorporate PI and stained red. The wild-type bacteria stained green indicating greater viability and a large proportion (95–97 %) of dead cells were found upon treatment with tobramycin in bacteria harbouring mutations in *pslC* and *pslI* (Fig. 3c, d). A 40–50 % reduction in biovolume and surface coverage was observed in the mutants after antibiotic treatment compared to the wild-type strain (Fig. 3e, f). This analysis confirmed that loss of glycosyl transferase results in an increase in tobramycin susceptibility in *P. aeruginosa.*

### The *pslC* and *pslI* mutation leads to an increase in swarming motility

**Fig. 4.**
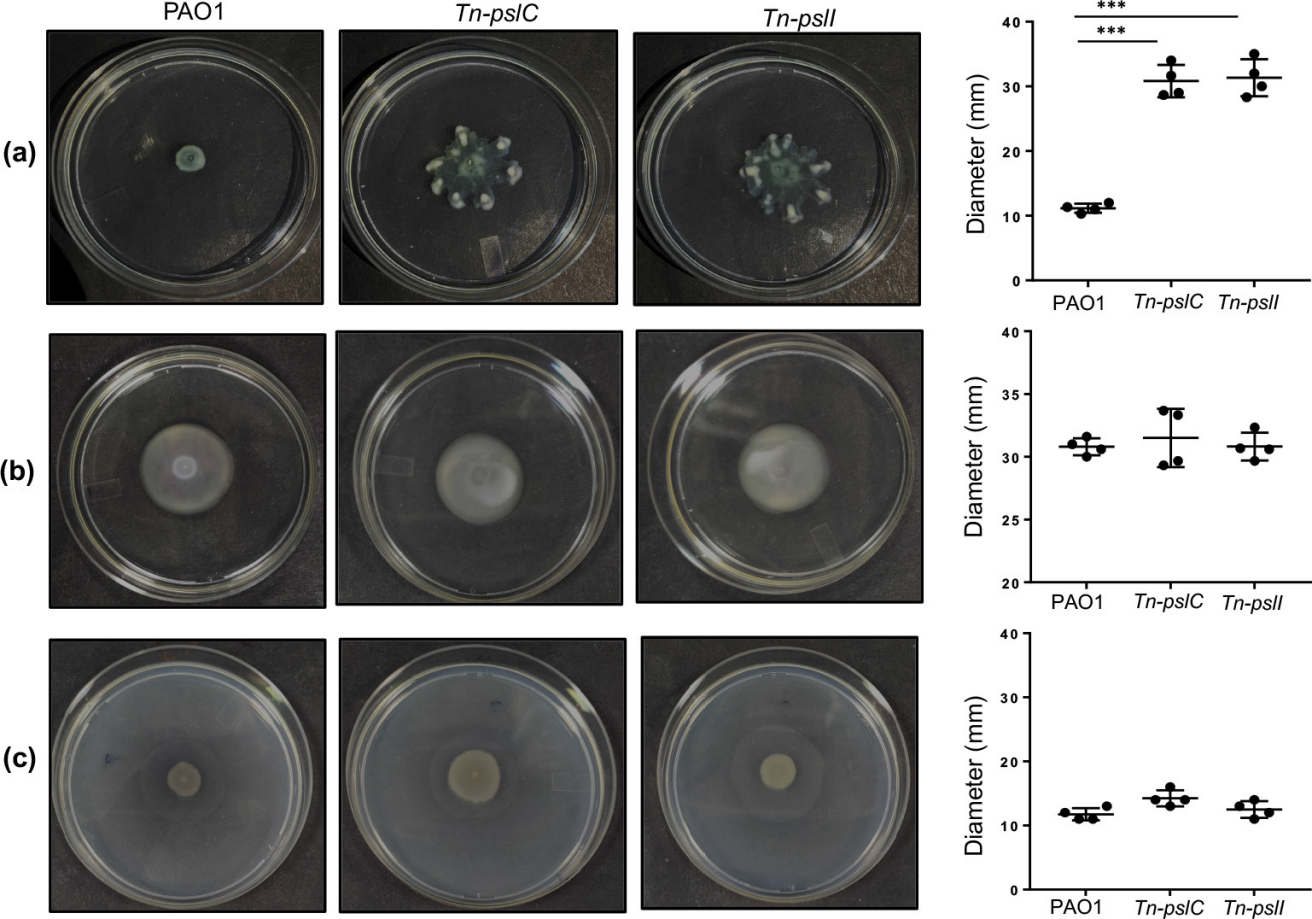
Motility of wild-type PAO1 and mutant *pslC* (*Tn-pslC*) and *pslI* (*Tn-pslI*) of *P. aeruginosa *. (**a**) Swarming (**b**) swimming and (**c**) twitching motility on agar plates observed after 14 h of incubation at 37 °C. The motility zone was evaluated by measuring the diameter of colonies on agar plates. The diameter of the motility zone of different strains is plotted. Data are averages calculated from three independent experiments in all cases. An unpaired *t*-test was performed to calculate probability values (***P≤0.001).

While swimming and swarming motility in bacteria are considered to be facilitated by flagella, twitching motility is related to pili [44]. Motility in *P. aeruginosa* is inversely connected to biofilm formation. To investigate if the mutation in *pslC* and *pslI* has any effect on motility of *P. aeruginosa *, the swimming, swarming and twitching motility of mutants and the wild-type was evaluated. The results demonstrated that while the wild-type PAO1 was impaired in swarming motility, there was a significant increase in swarming motility of the mutants (Fig. 4a). However, swimming and twitching motility showed no significant difference (Fig. 4b, c).

### The *pslC and pslI* mutation increases rhamnolipid formation in cells

**Fig. 5.**
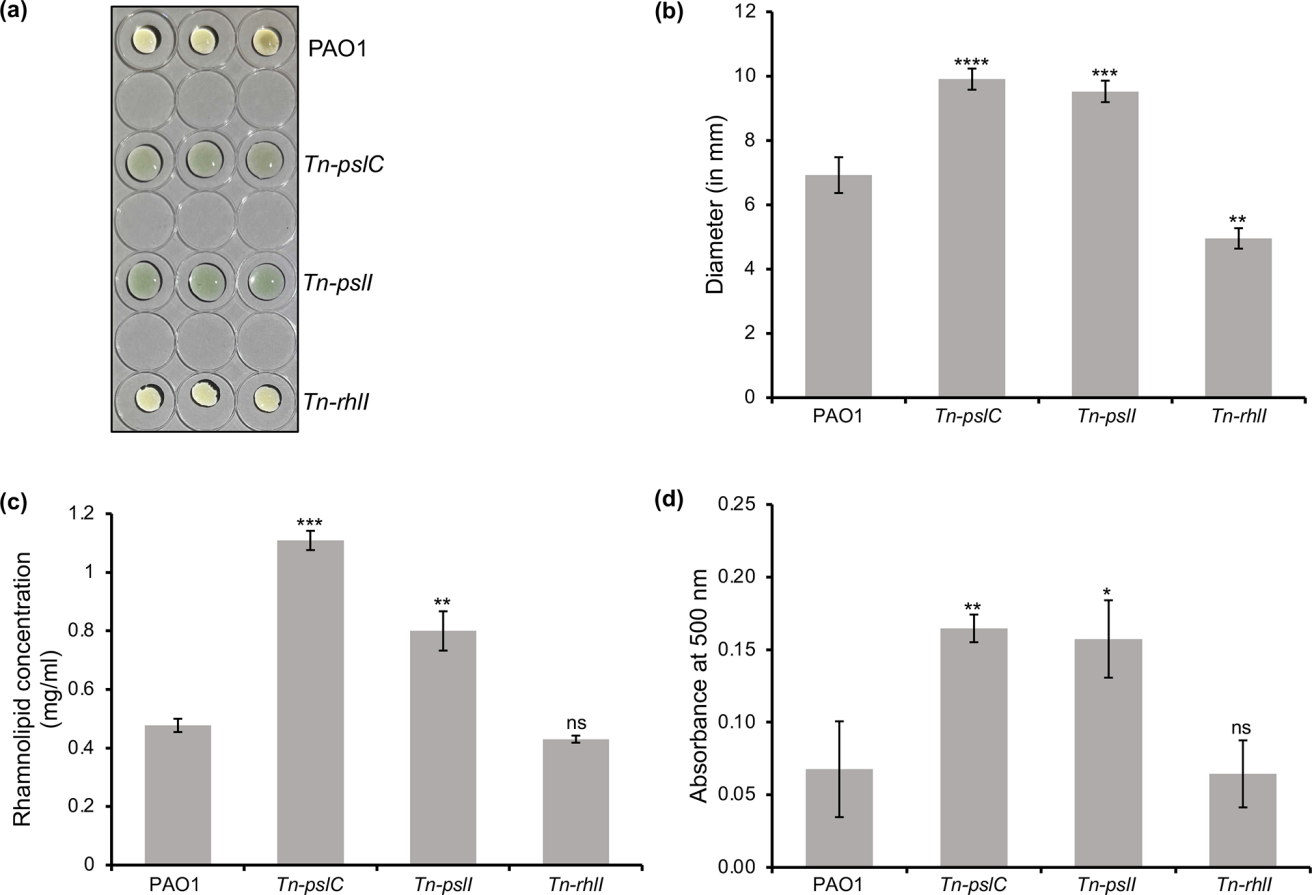
Effect of *psl* mutation on biosurfactant and pyocyanin formation. (**a**) Collapsed drops were observed in supernatant of *Tn-pslC* and *Tn-pslI* and bead-like drops were formed for wild-type PAO1 and *Tn-rhlI.* (**b**) Plot showing the diameter of droplets for the wild-type and mutant strains. (**c**) Quantification of rhamnolipid production in *P. aeruginosa* strains using an anthrone calorimetric assay. (**d**) Quantification of pyocyanin production by the *P. aeruginosa* strains performed by measuring the absorbance of a cell suspension at 500 nm. All experimental data are the mean of three independent experiments. An unpaired *t*-test was performed to evaluate probability values (*P ≤ 0.05; **P ≤ 0.01; ***P ≤ 0.001).

Swarming motility in bacteria is supported by hypersecretion of biosurfactants [45]. Rhamnolipids, the major biosurfactant in *P. aeruginosa *, help maintain the biofilm channels, formation of microcolonies, facilitate the 3-D mushroom-shaped structure and aid biofilm dispersion. Thus, we determined biosurfactant production using a drop collapse assay. This assay involves culturing bacteria for 48 h, and supernatant dropped on a hydrophobic surface collapses faster if the concentration of biosurfactant is high. It was observed that the supernatant of *Tn-pslC* and *Tn-pslI* showed high surfactant activity as suggested by collapsed drops compared to that of the parent strain (Fig. 5a, b). As a negative control, a drop collapse assay of supernatant of *Tn-rhlI* (transposon mutant of *rhlI*) was also performed. The drop of supernatant of *Tn-rhlI* was similar to the wild-type and no surfactant activity was observed. *P. aeruginosa* produces rhamnolipids as the major biosurfactant and we next extracted and quantified the rhamnolipids produced by mutants and wild-type strains (Fig. 5c). The results showed that the mutation in *pslC* and *pslI* led to a nearly two-fold increase in rhamnolipid formation compared to wild-type PAO1 while the *rhlI* mutant showed no difference.

### The mutation of *pslC and pslI* resulted in an increase in pyocyanin formation

One of the most well-studied pigments in *P. aeruginosa* is pyocyanin, an important virulence factor. We investigated pyocyanin production in the parental and mutant strains. The mutants showed higher pyocyanin production compared to wild-type PAO1 (Fig. 5d). Since growth of the three strains was comparable, this ruled out the possibility that the change in pyocyanin production was due to change in bacterial growth rate.

## Discussion

Biofilms are prevalent in the environment, clinics and industry. Exopolysaccharides are the most substantial components of biofilms. They serve as structural scaffolds for the attachment of other factors and to biotic and abiotic surfaces [32]. They also promote auto-aggregation which is crucial for biofilm formation and maintenance [15]. In addition, they form a protective layer that shields the bacteria from environmental threats, antimicrobials and the host defence system [46]. *P. aeruginosa* produces three different kinds of polysaccharides, Pel, Psl and alginate. Although the importance of exopolysaccharides in biofilm initiation and development is apparent, the exact mechanism of their synthesis remains poorly understood.

Psl is one of the predominant polysaccharides present in *P. aeruginosa* biofilms [13]. It has a helical distribution around the bacterial cell surface [32]. The Psl operon harbours 15 genes, 11 of which (*pslACDEFGHIJKL*) are required for the synthesis of Psl [15]. Mechanistic details of Psl synthesis are not well understood but are likely to resemble the lipid carrier-dependent biosynthesis of group 1 capsular polysaccharides in *E. coli* [47]. The biosynthetic machinery for Psl consists of an inner membrane-associated multiprotein complex which is coupled to its export. PslA bears similarity to WbaP, which suggests that it might be providing a site for assembly of an oligosaccharide repeat unit onto an isoprenoid lipid [16, 47]. PslB, a paralogue of WbpW (* P. aeruginosa *), serves as a bifunctional enzyme, with phosphomannose isomerase (PMI) and GDP-d-Man pyrophosphorylase (GMP) activities, and is involved in production of the sugar nucleotide precursor [14, 48]. On the other hand, PslD, a homologue of Wza, a translocase, is a periplasmic protein that facilitates the export of exopolysaccharide from bacterial cells [16, 49]. PslE shows similarity to Wzc and is predicted to function as the polysaccharide co-polymerase which interacts with PslA, PslD and PslG [16, 50, 51]. PslG is an endoglycosidase that localizes in the inner membrane and its hydrolytic activity has been shown to be important for Psl synthesis [51–53]. PslJ/PslK/PslL have extensive transmembrane regions and their function is not clear.

Psl contains d-mannose, d-glucose and l-rhamnose [54]. Sugar nucleotide precursors GDP-d-Man, UDP-d-Man and dTDP-l-Rha play crucial roles in Psl polysaccharide production [15]. Four enzymes, PslF, PslH, PslI and PslC, have glycosyltransferase fold and are predicted to be involved in the synthesis of glycans. Structural analysis carried out here and previously reveals that PslC and PslI might bind nucleotide sugar precursors which are transferred to sugar or non-sugar acceptor molecules resulting in the formation of repeating units of saccharides [16]. In fact, RfbN (rhamnosyl transferase) of *Salmonella typhimurium *, involved in LPS synthesis, was previously identified as the homologue of PslC [14]. Similarly, WbpY of *P. aeruginosa* shares homology with PslI [14]. Thus, mutation of *pslC* and *pslI* is expected to cause a reduction in biofilm biomass, biovolume and surface coverage as observed in this study. A reduction in biofilms has also been observed upon mutation of glycosyltransferases from other bacteria that have shown a similar phenotype [55, 56].

We also observed that the intracellular concentration of c-di-GMP diminished in the mutant strains. Since Psl functions as a signalling molecule for production of c-di-GMP, by stimulating diguanylate cyclases such as SadC and SiaD, a decrease in Psl levels is expected to affect this signalling resulting in lower levels of the secondary messenger [13]. Further, it has been demonstrated that low c-di-GMP induces expression of the QS regulator PqsR that activates the *rhl* and *pqs* systems. Activation of PqsR amplifies the production of rhamnolipids and pyocyanins [20]. Thus, the bacteria that are dispersed from biofilms have diminished levels of secondary messenger but have high levels of virulence factors.

Antibiotic therapy is the most effective treatment for *P. aeruginosa* infection, but biofilm formation renders the bacteria highly recalcitrant to antibiotics leading to treatment failures. The findings of the present study also revealed that mutation in either of the glycan processing enzymes, *pslC* or *pslI*, confers susceptibility to different classes of antibiotics in liquid cultures as well as in biofilm mode. Lowering the MBEC of the mutants is possibly due to the antibiotic being able to reach the bacterial cells which are otherwise sequestered by the polysaccharide layer. Alternatively, the role of these enzymes in glycosylation of the cell wall components needs to be explored.

Rhamnolipids are the major biosurfactants produced by *P. aeruginosa* [57]. They are glycolipids that contain one or two rhamnose molecules as the hydrophilic group linked to the fatty acids through glycosidic bonds. We observed nearly a two-fold elevation in rhamnolipid production upon mutation of putative glycosyl transferases. It has been suggested that the polysaccharides in bacteria are synthesized from a common intracellular pool of nucleotide sugar precursors, and therefore a surge in rhamnolipids may be due to increased availability of sugar precursors in the absence of Psl synthesis [45]. The presence of biosurfactants is also known to influence swarming in *P. aeruginosa* [58]. These are amphipathic molecules that reduce surface tensions. Thus, an increase in rhamnolipids also leads to an overall increase in swarming motility without affecting swimming and twitching motility of *P. aeruginosa *.

We also observed around a three-fold increase in pyocyanin production in the *Tn-pslC* and *Tn-pslI* strains. The synthesis of pyocyanin is under two operons in the PAO1 strain, *phz1* and *phz2,* and is regulated by QS regulators, RhlR and LasR. However, the mechanism underlying the increase in pigment formation remains unknown. Rhamnolipids and pyocyanin both serve as virulence factors and antimicrobials that provide an advantage to *P. aeruginosa* in competition with other microbes.

Overall, our work highlights the role of exopolysaccharide in facilitating biofilm formation and reversal of tolerance upon mutation of *pslC* and *pslI*. We suggest that the glycosyltransferases are potent targets for the development of novel anti-biofilms.

## Supplementary Data

Supplementary material 1
