# Erratum: Mutation of putative glycosyl transferases PslC and PslI confers susceptibility to antibiotics and leads to drastic reduction in biofilm formation in *Pseudomonas aeruginosa*

**DOI:** 10.1099/mic.0.001398

**Published:** 2023-10-10

**Authors:** Rohit Ruhal, Moumita Ghosh, Vineet Kumar, Deepti Jain

**Affiliations:** ^1^​ Transcription Regulation Lab, Regional Centre for Biotechnology, NCR Biotech Science Cluster, 3rd Milestone, Faridabad-Gurgaon Expressway, Faridabad, 121001, India

## Full-Text

In the published version of this article, there was an equal author contribution symbol on the first three authors.

Authors Rohit Ruhal and Moumita Ghosh should be the only authors with the equal author contribution symbol.

Previously, the author list appeared as below:

Rohit Ruhal†, Moumita Ghosh†, Vineet Kumar† and Deepti Jain*

The correct author list should have appeared as:

Rohit Ruhal†, Moumita Ghosh†, Vineet Kumar and Deepti Jain*

The version of record has been updated to reflect this and *Microbiology* apologises for any inconvenience.

