# Supplementary material for: Mutation of putative glycosyl transferases PslC and PslI confers susceptibility to antibiotics and leads to drastic reduction in biofilm formation in Pseudomonas aeruginosa
Source: Microbiology (Reading). 2023 Sep 13;169(9):001392. doi: 10.1099/mic.0.001392 (PMC10569066; doi:10.1099/mic.0.001392)
Supplement: Supplementary material 1 [file mic-169-1392-s001.pdf]

Mutation of putative glycosyl transferases PslC and PslI confers susceptibility to antibiotics and leads to drastic reduction in biofilm formation in *Pseudomonas aeruginosa*

Rohit Ruhal[#], Moumita Ghosh[#] and Deepti Jain[*]

Transcription Regulation Lab, Regional Centre for Biotechnology, NCR Biotech Science Cluster, 3rd Milestone, Faridabad-Gurgaon Expressway, Faridabad, 121001, India

#Contributed equally

*Correspondence- deepti@rcb.res.in

Table S1. Showing the DALI results for PslC

Score	PDB ID	RMSD	Identical hits	Percentage identity	Reference
20.9	6YV7	3.4	Mannosyl Transferase	16	26
18.5	5HEA	3.4	Putative glycosyl transferase	12	40
18.4	2Z86	2.7	Chondroitin synthase	19	27
17.4	1H7Q	2.9	Spore coat polysaccharide biosynthesis	15	41
17.1	4d11	3.3	GalNAc transferase	14	42

Table S2. Showing the DALI results for PslI

Score		PDB ID	RMSD	Identical hits	Percentage identity	Reference
32.7		6KIH	3.1	Sucrose phosphate synthase	20	[28]
30.7		2BIS	2.9	Glycogen Synthase from *Pyrococcus abyssi*	18	[43]
30.6		6N1X	3.0	Glycosyltransferase	17	[44]
29.4		3OKA	3.2	GDP-mannose-dependent alpha-(1-6)-phosphatidylinositol mannoside mannosyltransferase	18	[45]
28.9		4XSU	2.9	Alr3699 protein	18	[46]
